# Incidence and Prognosis of Ventilator-Associated Pneumonia in Critically Ill Patients with COVID-19: A Multicenter Study

**DOI:** 10.3390/jcm10040555

**Published:** 2021-02-03

**Authors:** Daniele Roberto Giacobbe, Denise Battaglini, Elisa Martina Enrile, Chiara Dentone, Antonio Vena, Chiara Robba, Lorenzo Ball, Michele Bartoletti, Irene Coloretti, Stefano Di Bella, Antonio Di Biagio, Iole Brunetti, Malgorzata Mikulska, Novella Carannante, Andrea De Maria, Laura Magnasco, Alberto Enrico Maraolo, Michele Mirabella, Giorgia Montrucchio, Nicolò Patroniti, Lucia Taramasso, Giusy Tiseo, Giacomo Fornaro, Fiorentino Fraganza, Luca Monastra, Erik Roman-Pognuz, Giacomo Paluzzano, Giuseppe Fiorentino, Antonio Corcione, Linda Bussini, Renato Pascale, Silvia Corcione, Tommaso Tonetti, Matteo Rinaldi, Marco Falcone, Emanuela Biagioni, Vito Marco Ranieri, Maddalena Giannella, Francesco Giuseppe De Rosa, Massimo Girardis, Francesco Menichetti, Pierluigi Viale, Paolo Pelosi, Matteo Bassetti

**Affiliations:** 1 Infectious Diseases Unit, San Martino Polyclinic Hospital-IRCCS for Oncology and Neurosciences, 16132 Genoa, Italy; chiaradentone@libero.it (C.D.); anton.vena@gmail.com (A.V.); antonio.dibiagio@hsanmartino.it (A.D.B.); m.mikulska@unige.it (M.M.); de-maria@unige.it (A.D.M.); lmagnasco90@gmail.com (L.M.); Michelemirabella90@gmail.com (M.M.); taramasso.lucia@gmail.com (L.T.); matteo.bassetti@unige.it (M.B.); 2 Anaesthesia and Intensive Care, San Martino Polyclinic Hospital-IRCCS for Oncology and Neurosciences, 16132 Genoa, Italy; Battaglini.denise@gmail.com (D.B.); elisa.martina.enrile@gmail.com (E.M.E.); kiarobba@gmail.com (C.R.); lorenzo.loryball@gmail.com (L.B.); brunettimed@gmail.com (I.B.); npatroniti@gmail.com (N.P.); ppelosi@hotmail.com (P.P.); 3 Department of Surgical Sciences and Integrated Diagnostics (DISC), University of Genoa, 16132 Genoa, Italy; 4 Infectious Diseases Unit, Department of Medical and Surgical Sciences, Policlinico Sant’Orsola, 40138 Bologna, Italy; m.bartoletti@unibo.it (M.B.); giacomo.fornaro@yahoo.it (G.F.); linda.bussini@gmail.com (L.B.); renpa@hotmail.it (R.P.); mat.rinaldi1989@gmail.com (M.R.); maddalena.giannella@unibo.it (M.G.); pierluigi.viale@unibo.it (P.V.); 5 Department of Anesthesia and Intensive Care, University Hospital of Modena, 41124 Modena, Italy; irenecoloretti@gmail.com (I.C.); emanuela.biagioni@gmail.com (E.B.); girardis@unimore.it (M.G.); 6 Clinical Department of Medical, Surgical and Health Science, Trieste University, 34127 Trieste, Italy; stefano932@gmail.com (S.D.B.); romanpognuz.erik@gmail.com (E.R.-P.); giacomo.paluzzano@gmail.com (G.P.); 7 Department of Health Sciences (DISSAL), University of Genoa, 16132 Genoa, Italy; 8 Emergenza Infettivologica PS, Cotugno Hospital, AORN dei Colli, 80131 Naples, Italy; carannantenovella@gmail.com; 9 First Division of Infectious Diseases, Cotugno Hospital, AORN dei Colli, 80131 Naples, Italy; albertomaraolo@mail.com; 10 Department of Surgical Sciences, University of Turin, 10126 Turin, Italy; g.montrucchio@gmail.com; 11 Unit of Anesthesia and Resuscitation 1, Department of Anesthesia, Intensive Care, and Emergency, Città della Salute e della Scienza, 10126 Turin, Italy; 12 Infectious Diseases Unit, Department of Clinical and Experimental Medicine, Azienda Ospedaliera Universitaria Pisana, University of Pisa, 56126 Pisa, Italy; tiseogiusy@gmail.com (G.T.); marco.falcone@unipi.it (M.F.); menichettifrancesco@gmail.com (F.M.); 13 Intensive Care Unit, Cotugno Hospital, AORN dei Colli, 80131 Naples, Italy; fiorentino.fraganza@ospedalideicolli.it (F.F.); lucamonastra@alice.it (L.M.); 14 Department of Anesthesia and Intensive Care Medicine; Azienda Sanitaria Universitaria Giuliano-Isontina (ASUGI), 34148 Trieste, Italy; 15 SubIntensiva Covid, Cotugno Hospital, AORN dei Colli, 80131 Naples, Italy; Giuseppefiorentino1@gmail.com; 16 Dipartimento Area Critica, AORN dei Colli, 80131 Naples, Italy; corcioneanto@gmail.com; 17 Department of Medical Sciences, Infectious Diseases, University of Turin, 10126 Turin, Italy; corcione.silvia@gmail.com (S.C.); francescogiuseppe.derosa@unito.it (F.G.D.R.); 18 Intensive Care Unit, Department of Medical and Surgical Sciences, Policlinico Sant’Orsola, 40138 Bologna, Italy; tommaso.tonetti@unibo.it (T.T.); m.ranieri@unibo.it (V.M.R.)

**Keywords:** VAP, COVID-19, SARS-CoV-2, coronavirus, ventilation

## Abstract

The primary objective of this multicenter, observational, retrospective study was to assess the incidence rate of ventilator-associated pneumonia (VAP) in coronavirus disease 2019 (COVID-19) patients in intensive care units (ICU). The secondary objective was to assess predictors of 30-day case-fatality of VAP. From 15 February to 15 May 2020, 586 COVID-19 patients were admitted to the participating ICU. Of them, 171 developed VAP (29%) and were included in the study. The incidence rate of VAP was of 18 events per 1000 ventilator days (95% confidence intervals [CI] 16–21). Deep respiratory cultures were available and positive in 77/171 patients (45%). The most frequent organisms were *Pseudomonas aeruginosa* (27/77, 35%) and *Staphylococcus aureus* (18/77, 23%). The 30-day case-fatality of VAP was 46% (78/171). In multivariable analysis, septic shock at VAP onset (odds ratio [OR] 3.30, 95% CI 1.43–7.61, *p* = 0.005) and acute respiratory distress syndrome at VAP onset (OR 13.21, 95% CI 3.05–57.26, *p* < 0.001) were associated with fatality. In conclusion, VAP is frequent in critically ill COVID-19 patients. The related high fatality is likely the sum of the unfavorable prognostic impacts of the underlying viral and the superimposed bacterial diseases.

## 1. Introduction

Severe acute respiratory syndrome coronavirus-2 (SARS-CoV-2) is the causative agent of coronavirus disease 2019 (COVID-19). Most clinically detectable infections are mild to moderate, but cases of severe pneumonia requiring intensive care unit (ICU) admission may be observed [1,2,3,4].

The clinical presentation of COVID-19 pneumonia includes fever, leukocytosis, severe hypoxemia, bilateral infiltrates, and multisystemic inflammatory syndrome with possible multiorgan failure (MODS-CoV-2) [5,6]. Some COVID-19 patients admitted to the ICU may require mechanical ventilation for a long time, putting them at risk of developing bacterial superinfections, including ventilator-associated pneumonia (VAP), that may contribute to unfavorably influencing prognosis [7,8,9]. However, a clear picture of the true incidence rate, spectrum of causative agents, and prognostic factors of VAP in COVID-19 patients, which may help in improving its management, is still unavailable.

The primary objective of this observational, multicenter study was to assess the incidence rate of VAP in COVID-19 patients. The secondary objective was to assess predictors of 30-day case-fatality of VAP in COVID-19 patients.

## 2. Materials and Methods

### 2.1. Study Design and Setting

The present multicenter, observational, retrospective study was conducted in 11 intensive care units (ICU) across 9 centers in Italy (see Supplementary Materials Table S1 for details) from 15 February 2020 to 15 May 2020. All patients with COVID-19 who developed VAP during ICU stay were included in the study. Ventilator days of both VAP and non-VAP COVID-19 patients were also collected for calculating the incidence rate of VAP. The primary study endpoint was the incidence rate of VAP. Secondary study endpoints were: (i) 30-day case-fatality of VAP; (ii) 30-day case-fatality of bronchoalveolar lavage fluid (BALF)-positive VAP.

The collection of anonymized data for the present study was approved by the Ethics Committee of the coordinating center (Liguria Region Ethics Committee, registry number 163/2020), and specific informed consent was waived due to the retrospective nature of the study. The other participating centers followed the local ethical requirements.

### 2.2. Definitions

The diagnosis of COVID-19 was made in presence of at least one positive real-time polymerase chain reaction (RT-PCR) test for SARS-CoV-2 on respiratory specimen/s (nasopharyngeal swab, sputum, and/or lower respiratory tract specimens). VAP was defined as new or changing chest X-ray infiltrate/s occurring more than 48 h after initiation of invasive mechanical ventilation, plus both of the following: (i) new onset of fever (body temperature ≥ 38 °C)/hypothermia (body temperature ≤ 35 °C) and/or leukocytosis (total peripheral white blood cell count ≥ 10,000 cells/μL)/leukopenia (total WBC count ≤ 4500 cells/μL)/ > 15% immature neutrophils; (ii) new onset of suctioned respiratory secretions and/or need for acute ventilator support system changes to enhance oxygenation [10]. BALF-positive VAP was defined as VAP with a positive BALF culture for bacterial respiratory pathogens. Ventilator days were defined as days with an invasive device in the airways, including tracheostomy.

### 2.3. Data Collection

Anonymized demographic and clinical data were collected using REDCap (Research Electronic Data Capture), a secure, web-based application designed to support data capture for research studies [11]. Data were collected for first VAP episodes. The following demographic and clinical data were collected: age in years; gender; body mass index; diabetes mellitus; hypertension; smoking; respiratory disease (defined as asthma or chronic obstructive pulmonary disease); end-stage renal disease (defined as estimated glomerular filtration rate <15 mL/min/1.73 m^2^); moderate-to-severe liver failure (defined as compensated or decompensated liver cirrhosis); neurologic disease (defined as at least one of the following: epilepsy, Alzheimer disease or other dementias, cerebrovascular diseases including stroke, migraine and other headache disorders, multiple sclerosis, Parkinson’s disease, infections of the nervous system, brain tumors, traumatic disorders of the nervous system due to head trauma, and neurological disorders as a result of malnutrition); solid cancer; hematological malignancy; human immunodeficiency virus infection; previous antibiotic therapy (within 30 days before VAP onset); previous anti-inflammatory treatments (within 30 days before VAP onset); days of invasive ventilation before VAP; sequential organ failure assessment (SOFA) score [12]; tracheostomy before VAP. The following variables were collected as they were at VAP onset: presence of septic shock (defined according to sepsis-3 criteria [13]); presence of at least mild acute respiratory distress syndrome (ARDS) [14]; presence of acute kidney injury according to RIFLE criteria [15]; need for hemodialytic therapy; need for extracorporeal membrane oxygenation (ECMO); presence of thrombotic or hemorrhagic disorders; bronchoscopy with BALF collection performed at VAP onset (yes/no) and related BALF culture results; concomitant bloodstream infection (BSI). The following variables were also collected regarding the management of VAP: administration of IgM-enriched intravenous immunoglobulins; use of cytokine blood filter/s; timing of antibiotic therapy; appropriateness of antibiotic therapy (measured in the subgroup of patients with BALF-positive VAP and defined as therapy with at least one agent displaying in vitro activity against the given BALF isolate/s (and against blood cultures isolate/s in patients with concomitant BSI)). Isolates were identified by automated biochemical-based phenotypic identification systems or MALDI-TOF, according to the standard procedures of the different local microbiology laboratories. Susceptibility test results were obtained using automated dilution methods and interpreted according to European Committee on Antimicrobial Susceptibility Testing (EUCAST) breakpoint tables (version 10.0, 2020; http://www.eucast.org).

### 2.4. Sample Size Calculation

The number of participating centers was selected in order to guarantee, based on local estimates, a minimum sample size of 4000 ventilator days. This was considered an acceptable compromise between feasibility and generalizability of study results with regard to the primary descriptive endpoint (incidence rate of VAP in COVID-19 patients). Indeed, by assuming normal distribution of the measure of interest, a sample size of 4000 ventilator days would have guaranteed a maximum margin of error (95% confidence interval [CI]) of ±5 events for an expected incidence rate of ≤20 VAP episodes per 1000 ventilator days.

### 2.5. Statistical Analysis

The primary study aim was to assess the incidence rate of VAP in COVID-19 patients, that was calculated as the number of events per 1000 ventilator days. Actual confidence intervals of the incidence rate estimate were calculated by means of the exact mid-p test [16]. For the secondary study analysis (assessment of predictors of 30-day case fatality), predefined demographic and clinical variables were first tested for their association with the outcome in univariable logistic regression models. Then, factors potentially associated with 30-day case-fatality in univariable analysis (*p* < 0.10) were included in a multivariable logistic regression model (model A). Variables related to antibiotic therapy, which we deemed as clinically relevant (as they are modifiable interventions), were included in model A, independent of their *p*-value in univariable comparisons. No stepwise procedure was adopted. All variables included in model A were also tested for their association with 30-day case fatality in an additional multivariable, generalized, linear mixed model (model B, with center as a random effect and logit as the link function). A pre-planned subgroup analysis of factors associated with 30-day case fatality was conducted in patients with BALF-positive VAP. A descriptive comparison of 30-day case fatality in patients who did not undergo bronchoscopy and patients with positive BALF culture was performed with the Kaplan–Meier method and the log-rank test, and with the day of VAP onset as the time of origin. The analyses were performed using R Statistical Software version 3.5.2 (R Foundation for Statistical Computing, Vienna, Austria). Results of the mixed model were obtained by using the glmer function in the lme4 package for R Statistical Software.

## 3. Results

During the study period, 586 patients with severe COVID-19 infections required invasive mechanical ventilation and were admitted to the participating ICU, for a total of 9416 ventilator-days. Overall, 171/586 (29%) patients were diagnosed with VAP. The median time elapsed from ICU admission to VAP development was of 10 days (interquartile range 6–17). The incidence rate of VAP was of 18 events per 1000 ventilator days (95% CI 16–21). Five additional patients were included in the electronic data capture systems, but they did not fulfill criteria for inclusion (Supplementary Materials Figure S1).

The demographic and clinical characteristics of COVID-19 patients with VAP are shown in Table 1. Their median age was 64 years (interquartile range (IQR) 57–71) and 80% were males (137/171). The most frequent comorbid conditions were hypertension (109/171; 64%) and diabetes mellitus (39/171; 23%). Before developing VAP, most patients received antibiotic treatment (162/171; 95%), mostly cephalosporins (88/171; 52%) and macrolides (78/171; 46%). As many as 159/171 (93%) patients were previously treated with chloroquine or hydroxychloroquine, whereas 108/171 (63%) and 109/171 (64%) received steroids and anti-interleukin 6 (IL-6) monoclonal antibodies, respectively. BALF specimens were obtained in 79/171 cases (46%), with culture being positive in 77/79 of them (97%). The most frequently isolated organisms were *Pseudomonas aeruginosa* (27/77, 35%), *Staphylococcus aureus* (18/77, 23%), and *Klebsiella pneumoniae* (15/77, 19%). Details on isolated organisms are available in Supplementary Materials Table S2. Overall, 8/77 (10%) and 25/77 (32%) of BALF cultures yielded methicillin-resistant *S. aureus* and carbapenem-resistant Gram-negative bacteria, respectively. Empirical antibiotic treatment was administered within 24 h from VAP onset in 125/171 patients (73%), whereas appropriate antibiotic treatment (assessed in the subgroup of patients with positive BALF culture) was administered within 24 h from VAP onset in 45/77 cases (58%). A descriptive comparison of the demographic and clinical characteristics of patients with and without availability of BALF cultures is available in Supplementary Materials Table S3.

**Figure 1:**
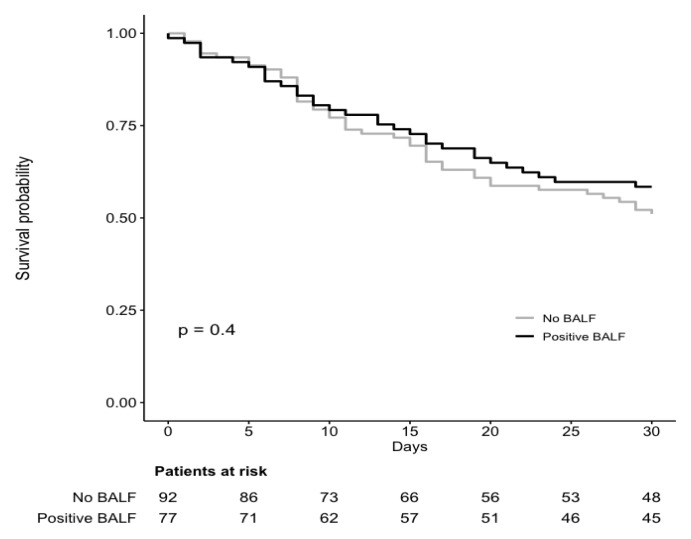
Survival in critically ill COVID-19 patients with VAP. Comparison of survival in patients who did not underwent bronchoscopy and patients with positive culture of bronchoalveolar lavage fluid (BALF) at ventilator-associated pneumonia (VAP) onset. The time of origin was set at the day of VAP onset. *p*-value is from log-rank test. Two patients with negative BALF cultures were excluded from this analysis.

The 30-day case-fatality of VAP was 46% (78/171). Table 2 and Table 3 show the results of univariable and multivariable analyses, respectively, of factors associated with 30-day fatality. In univariable analysis, higher SOFA score, septic shock at VAP onset, ARDS at VAP onset, AKI at VAP onset, hemodialytic therapy at VAP onset, and ECMO at VAP onset were unfavorably associated with the outcome, whereas previous treatment with anti-IL-6 receptor monoclonal antibodies and tracheostomy before VAP were associated with reduced 30-day case fatality. In multivariable analysis (model A), only septic shock at VAP onset (odds ratio (OR) 3.30, 95% CI 1.43–7.61, *p* = 0.005), and ARDS at VAP onset (OR 13.21, 95% CI 3.05–57.26, *p* < 0.001) retained an independent association with the outcome. As shown in Table 3, the results of the additional multivariable model with center as a random effect (model B) were in line with those of model A. The 30-day case-fatality of BALF culture-positive VAP was 42% (32/77). Results of univariable and multivariable analyses of factors associated with 30-day case-fatality in this subgroup are reported in detail in Supplementary Materials Tables S4 and S5. In the multivariable model, ARDS at VAP onset showed an independent association with 30-day case-fatality (Table S5). Similar Kaplan–Meier curves were observed for 30-day case fatality in patients who did not undergo bronchoscopy vs. patients with positive BALF cultures (Figure 1).

## 4. Discussion

In our multicenter cohort, the incidence rate of VAP in critically ill patients with COVID-19 was as high as 18 events per 1000 ventilator days in ICU, with 30-day fatality of VAP being as high as 46%. The most frequent causative organism was *P. aeruginosa*, followed by *S. aureus.*

The incidence rate of VAP we reported in COVID-19 critically ill patients is among the highest when compared to that of 1 to 19 episodes per 1000 ventilator days reported in non-COVID-19 patients [17,18,19,20,21]. There are different reasons that may explain this high incidence rate we registered. On the one hand, a truly increased risk of VAP in COVID-19 patients (which is in line with the high incidence rate of 28 episodes per 1000 ventilator-days registered in a recent UK study and with the high reported prevalence of 58% in a large cohort of 4244 critically ill patients with COVID-19 [22,23]), might be explained by different reasons: (i) a potential increased predisposition to bacterial superinfection, on the top of lung damage caused by COVID-19; (ii) the virus-related immunosuppressive effect with deep lymphopenia; (iii) the potential concomitant anti-inflammatory or immunosuppressive effect of steroids and biologic agents (e.g., anti-IL-6 receptor monoclonal antibodies) [24,25]. On the other hand, supporting instead a possible artefactual increase of the registered VAP incidence rate, we may have included some patients diagnosed with VAP who in reality did not have VAP, since we used a broad definition of VAP that is generally used for enrollment in clinical trials rather than for epidemiological purposes. This was done because of the non-negligible frequency of lack of microbiological data, that would have rendered unreliable other more specific definitions of VAP. Indeed, achieving etiological diagnosis of VAP in COVID-19 patients remains difficult for at least two major reasons: (i) there could be a reduced propensity to collect deep respiratory specimens (BALF), owing to the risks either of worsening hypoxemia or of SARS-CoV-2 transmission to healthcare workers; (ii) information from less invasive specimens (e.g., from endotracheal aspirate) may not allow to easily differentiate between airway colonization or pulmonary bacterial superinfection in COVID-19 patients, even when using traditional quantitative thresholds [5]. In addition, either presentation or worsening of COVID-19 pneumonia share many features with VAP, such as fever, hypoxemia, consolidative infiltrates, and alterations in inflammatory markers [5]. For all these reasons, there could be a risk of VAP overdiagnosis in critically ill COVID-19 patients. However, it should also be noted that our numerator for the calculation of the incidence rate was made only of first VAP episodes. Therefore, since some patients may have experienced more than one VAP episode, we also cannot exclude an underestimation of the true incidence rate of VAP in critically ill COVID-19 patients. With regard to organisms isolated from deep respiratory specimens in patients with VAP in our series, the higher frequency of Gram-negative bacteria we registered is in line with recent data from other countries [22,26,27].

In this study, we also assessed predictors of 30-day case fatality in COVID-19 patients with VAP. The associations of both ARDS and septic shock with fatality likely testify to the well-known unfavorable prognostic effect of severe acute conditions at VAP presentation [28], which is also confirmed in our additional mixed model accounting for variability across centers. Furthermore, the unfavorable prognostic effect of ARDS is in line with the results observed in the subgroup of patients with BALF-positive VAP. The prognosis may be influenced by two concomitant diseases (COVID-19 and VAP superinfection). Consequently, it cannot be excluded that the course of COVID-19 may exert a modifying effect on the prognostic impact of potential predictors of unfavorable VAP prognosis explored in this study. In this regard, we did not find an association between early antibiotic therapy and reduced fatality, and also between early appropriate antibiotic therapy and reduced fatality in the subgroup of patients with BALF-positive VAP, differently from what observed in classical ICU populations [28]. Although this result may merely depend on the low power of our analyses (especially in the subgroup of patients with BALF-positive VAP in which we were able to assess early appropriate therapy, considering that the direction of the prognostic effect, although not statistically significant, was towards reduced fatality), it is also plausible that the interfering effect of the viral disease may play an important role as confounder. Notably, there could also be a relevant background noise played by the already well-known difficulties in clearly deciphering the attributable mortality of VAP in ICU populations in general [20].

The present study has some important limitations. First, it was observational and retrospective, which inherently implies some risks of selection and information biases. Nonetheless, at least for the latter, we tried to minimize them by employing a real-time review of inserted data by a dedicated central investigator (DB), with rapid generation of pertinent queries to be resolved by local investigators. Second, we were unable to retrospectively collect precise quantitative (in terms of colony forming units [CFU]/mL) rather than qualitative information from positive BALF cultures, as well as quantitative data from endotracheal aspirate cultures (for this reason we ultimately decided to include deeper [BALF] and not endotracheal cultures for subgroup analysis in the attempt to obtain a distribution of bacteria possibly closer to infection than colonization). We acknowledge this was an arbitrary decision in order to identify what we thought was the best subgroup for a more plausible estimation of etiological diagnosis considering the limited available data collected during routine practice in the first months of the COVID-19 pandemic. In this regard, the lack of many microbiological data certainly remains a major limitation of the present study, since all of this lacking microbiological information could have been exploited either for a more precise selection of the subgroup of patients with microbiological diagnosis of VAP or for exploring the possible prognostic effects of different CFU/mL counts. Third, in line with the used VAP definition [10], and besides the risk of incidence overestimation as described in the previous paragraphs, there is also the major limitation that we only collected data on bacteria, and not on other organisms that may cause deep respiratory infections in critically ill COVID-19 patients (e.g., COVID-19-associated pulmonary aspergillosis (CAPA) [29,30]). Although CAPA has specific, proposed diagnostic criteria that would have required dedicated and systematic data collection from all patients for a reliable incidence picture [30], it is likely that some patients in the present study also had CAPA, thus, we cannot exclude an independent prognostic effect of CAPA as unmeasured confounding in the analysis of prognostic predictors. Fourth, there was a possible inclusion of too many variables in our multivariable models, although we ultimately preferred not to remove potential explanatory variables on the basis of stepwise selection in view of the purely exploratory nature of our analysis [31,32]. Fifth, although data of critically ill patients without VAP was not collected since the aim of this study was not to assess predictors of VAP, this precluded a descriptive comparison of crude fatality in COVID-19 patients with and without VAP. Finally, the lack of information regarding both patient-level and center-level VAP prevention systems does not allow to infer their contribution to the risk of VAP (and, in turn, to VAP incidence) in the present study.

## 5. Conclusions

VAP may be frequent in critically ill COVID-19 patients, but its clinical diagnosis remains difficult. The high 30-day case fatality of VAP we observed likely represents the sum of the prognostic effects of the underlying viral and the superimposed bacterial diseases. Further investigation is needed to precisely characterize the relative contribution of these effects and further improve our therapeutic approach to both COVID-19 and superimposed VAP.

## Figures and Tables

**Table 1:** Characteristics of 171 critically ill COVID-19 patients who developed ventilator-associated pneumonia (VAP).

Variable	No. of Patients 171 (100)
Demographics	
Age in years, median (IQR)	64 (57–71)
Male gender	137 (80)
BMI in kg/m^2^, median (IQR)	28 (25–30)
Baseline comorbidities	
Diabetes mellitus	39 (23)
Hypertension	109 (64)
Smoking/respiratory disease	27 (16)
End-stage renal disease	10 (6)
Moderate/severe liver failure	3 (2)
Neurologic disease	6 (4)
Solid cancer	11 (6)
Hematological malignancy	4 (2)
HIV infection	0 (0)
Previous antibiotic therapy *	
Any	162 (95)
Semisynthetic penicillins	76 (44)
Cephalosporins	88 (52)
Carbapenems	25 (15)
Polymyxins	2 (1)
Glycopeptides	10 (6)
Oxazolidinones	38 (22)
Macrolides	78 (46)
Fluoroquinolones	5 (3)
Aminoglycosides	0 (0)
Previous anti-inflammatory therapy *	
Steroids	108 (63)
NSAIDs	40 (23)
Chloroquine/Hydroxychloroquine	159 (93)
Anti-IL-6 receptor monoclonal antibodies	109 (64)
Monoclonal IL-1 receptor antagonists	5 (3)
VAP characteristics	
Days of invasive ventilation before VAP, median (IQR)	9 (5–15)
SOFA score at VAP onset, median (IQR)	7 (4–9)
Tracheostomy before VAP	49 (29)
Presence of septic shock at VAP onset	80 (47)
Presence of ARDS at VAP onset	132 (77)
Presence of AKI at VAP onset	41 (24)
Need for hemodialytic therapy at VAP onset	17 (10)
Need for ECMO at VAP onset	13 (8)
Presence of coagulative disorders at VAP onset	
None	132 (77)
Thrombotic	20 (12)
Hemorrhagic	17 (10)
Both	2 (1)
Bronchoscopy with BALF collection at VAP onset	
Not performed	92 (54)
Negative BALF culture	2 (1)
Positive BALF culture	77 (45)
BSI at VAP diagnosis **	76 (44)
VAP treatment	
Antibiotic treatment within 24 h of VAP onset	125 (73)
IgM-enriched intravenous immunoglobulins	10 (6)
Cytokine blood filtration	18 (11)
Outcome	
30-day case fatality	78 (46)

Results are reported as number of patients (%) unless otherwise indicated. AKI, acute kidney injury; ARDS, acute respiratory distress syndrome; BALF, bronchoalveolar lavage fluid; BMI, body mass index; BSI, bloodstream infection; COVID-19, coronavirus disease 2019; ECMO, extracorporeal membrane oxygenation; HIV, human immunodeficiency virus; IQR, interquartile range; NSAIDs, nonsteroidal anti-inflammatory drugs; SOFA, sequential organ failure assessment; VAP, ventilator-associated pneumonia. * Within 30 days; ** 33 BSI episodes were registered in patients with no BALF specimens or negative BALF cultures (33/94, 35%), whereas 43 BSI episodes were registered in patients with positive BALF cultures (43/77, 56%). In patients with positive BALF cultures, 17/43 BSI episodes (40%) were caused by the same pathogens isolated from BALF cultures, 21/43 (49%) were caused by different pathogens from those isolated from BALF cultures, and 5/43 (12%) were caused both by the same pathogens isolated from BALF cultures and from other pathogens.

**Table 2:** Univariable analysis of factors associated with 30-day case fatality in critically ill COVID-19 patients with VAP.

Variable	Non-Survivors 78 (46)	Survivors 93 (54)	OR (95% CI)	*p*
Demographics				
Age in years, median (IQR)	62 (57–72)	66 (57–71)	1.00 (0.97–1.03)	0.930
Male gender	63 (81)	74 (80)	1.08 (0.51–2.30)	0.845
BMI in kg/m^2^, median (IQR)	28 (25–31)	27 (25–30)	1.00 (0.99–1.02)	0.812
Baseline comorbidities				
Diabetes mellitus	20 (26)	19 (20)	1.34 (0.66–2.75)	0.419
Hypertension	49 (63)	60 (65)	0.93 (0.50–1.74)	0.818
Smoking/respiratory disease	15 (19)	29 (31)	0.53 (0.26–1.07)	0.077
End-stage renal disease	6 (8)	4 (4)	1.85 (0.50–6.82)	0.353
Moderate/severe liver failure	2 (3)	1 (1)	2.42 (0.22–27.22)	0.474
Neurologic disease	2 (3)	4 (4)	0.59 (0.10–3.29)	0.543
Solid cancer	4 (5)	7 (8)	0.66 (0.19–2.36)	0.527
Hematological malignancy	3 (4)	1 (1)	3.68 (0.38–36.11)	0.263
HIV infection	0 (0)	0 (0)	-	-
Previous antibiotic therapy				
Any	72 (92)	90 (97)	0.40 (0.10–1.66)	0.206
Semisynthetic penicillins	36 (46)	40 (43)	1.14 (0.62–2.08)	0.680
Cephalosporins	35 (45)	53 (57)	0.61 (0.34–1.13)	0.115
Carbapenems	11 (14)	14 (15)	0.93 (0.39–2.18)	0.861
Polymyxins *	0 (0)	2 (2)	0.23 (0.00–2.92)	0.444
Glycopeptides	6 (8)	4 (4)	1.85 (0.50–6.82)	0.353
Oxazolidinones	18 (23)	20 (22)	1.10 (0.53–2.26)	0.806
Macrolides	33 (42)	45 (48)	0.78 (0.43–1.43)	0.427
Fluoroquinolones	3 (4)	2 (2)	1.82 (0.30–11.18)	0.518
Aminoglycosides	0 (0)	0 (0)	-	-
Previous anti-inflammatory therapy				
Steroids	51 (65)	57 (61)	1.19 (0.64–2.23)	0.581
NSAIDs	13 (17)	27 (29)	0.49 (0.23–1.03)	0.060
Chloroquine/Hydroxychloroquine	71 (91)	88 (95)	0.58 (0.18–1.89)	0.364
Anti-IL-6 receptor monoclonal antibodies	43 (55)	66 (71)	0.50 (0.27–0.95)	0.033
Monoclonal IL-1 receptor antagonists	3 (4)	2 (2)	1.82 (0.30–11.18)	0.518
VAP characteristics				
Days of invasive ventilation before VAP, median (IQR)	9 (5–14)	10 (5–17)	0.99 (0.95–1.02)	0.487
SOFA score at VAP onset, median (IQR)	8 (5–11)	6 (3–8)	1.21 (1.09–1.33)	<0.001
Tracheostomy before VAP	16 (21)	33 (36)	0.47 (0.23–0.94)	0.033
Presence of septic shock at VAP onset	48 (62)	32 (34)	3.05 (1.63–5.70)	<0.001
Presence of ARDS at VAP onset	74 (95)	58 (62)	11.16 (3.75–33.21)	<0.001
Presence of AKI at VAP onset	25 (32)	16 (17)	2.27 (1.11–4.66)	0.025
Need for hemodialytic therapy at VAP onset	12 (15)	5 (5)	3.20 (1.08–9.53)	0.037
Need for ECMO at VAP onset	11 (14)	2 (2)	7.47 (1.60–34.82)	0.010
Presence of coagulative disorders at VAP onset *				0.188
None	59 (76)	73 (79)	Ref.	
Thrombotic	6 (8)	14 (15)	0.55 (0.19–1.44)	
Hemorrhagic	11 (14)	6 (7)	2.19 (0.81–6.41)	
Both	2 (3)	0 (0)	6.18 (0.49–858.32)	
BALF collection for culture at VAP onset	33 (42)	46 (50)	0.75 (0.41–1.37)	0.350
BSI at VAP onset	33 (42)	43 (46)	0.85 (0.47–1.56)	0.607
VAP treatment				
Antibiotic treatment within 24 h of VAP onset	57 (73)	68 (73)	1.00 (0.51–1.97)	0.995
IgM-enriched intravenous immunoglobulins	7 (9)	3 (3)	2.96 (0.74–11.85)	0.126
Cytokine blood filtration	8 (10)	10 (11)	0.95 (0.36–2.53)	0.916

Results are reported as number of patients (%) unless otherwise indicated. AKI, acute kidney injury; ARDS, acute respiratory distress syndrome; BALF, bronchoalveolar lavage fluid; BMI, body mass index; BSI, bloodstream infection; CI, confidence intervals; COVID-19, coronavirus disease 2019; ECMO, extracorporeal membrane oxygenation; HIV, human immunodeficiency virus; IQR, interquartile range; NSAIDs, nonsteroidal anti-inflammatory drugs; OR, odds ratio; SOFA, sequential organ failure assessment; VAP, ventilator-associated pneumonia. * Standard logistic regression model not converging for this variable. Results for are from univariable logistic regression with Firth’s correction.

**Table 3:** Multivariable analysis of independent predictors of 30-day case fatality in critically ill COVID-19 patients with VAP.

**Model A (AIC 201.9)**	**OR (95% CI)**	***p***
Smoking/respiratory disease	0.57 (0.24–1.38)	0.213
Previous NSAIDs	1.14 (0.38–3.42)	0.811
Previous anti-IL-6 receptor monoclonal antibodies	0.68 (0.32–1.45)	0.316
Tracheostomy before VAP	0.50 (0.22–1.16)	0.108
SOFA score at VAP onset	1.07 (0.93–1.24)	0.326
Presence of septic shock at VAP onset	3.30 (1.43–7.61)	0.005 *
Presence of ARDS at VAP onset	13.21 (3.05–57.26)	<0.001 *
Presence of AKI at VAP onset	0.62 (0.23–1.66)	0.340
Need for hemodialytic therapy at VAP onset	3.11 (0.79–12.2)	0.104
Need for ECMO at VAP onset	3.19 (0.55–18.56)	0.197
Antibiotic treatment within 24 h of VAP onset	0.67 (0.27–1.63)	0.337
Model B ** (AIC 203.0)	**OR (95% CI)**	***p***
Smoking/respiratory disease	0.54 (0.22–1.34)	0.185
Previous NSAIDs	1.44 (0.40–5.20)	0.581
Previous anti-IL-6 receptor monoclonal antibodies	0.66 (0.29–1.49)	0.317
Tracheostomy before VAP	0.47 (0.19–1.15)	0.100
SOFA score at VAP onset	1.12 (0.93–1.34)	0.238
Presence of septic shock at VAP onset	3.22 (1.33–7.80)	0.010 *
Presence of ARDS at VAP onset	12.71 (2.74–58.89)	0.001 *
Presence of AKI at VAP onset	0.64 (0.23–1.82)	0.404
Need for hemodialytic therapy at VAP onset	3.08 (0.73–13.00)	0.126
Need for ECMO at VAP onset	2.35 (0.34–16.28)	0.387
Antibiotic treatment within 24 h of VAP onset	0.66 (0.26–1.66)	0.375

Results are reported as number of patients (%) unless otherwise indicated. AIC, Akaike information criterion; AKI, acute kidney injury; ARDS; acute respiratory distress syndrome; CI, confidence intervals; COVID-19, coronavirus disease 2019; ECMO, extracorporeal membrane oxygenation; OR, odds ratio; SOFA, sequential organ failure assessment; VAP, ventilator-associated pneumonia. * *p* < 0.05; ** Model B included center as a random effect.

## Data Availability

The data presented in this study are available on reasonable request from the corresponding author.

## Supplementary Materials

The following are available online at https://www.mdpi.com/2077-0383/10/4/555/s1, Figure S1: flow-chart of the patient inclusion process; Table S1: list of participating centers; Table S2: isolates from BALF cultures; Table S3: descriptive comparison of the demographic and clinical characteristics of patients with and without BALF culture; Table S4: univariable analysis of factors associated with 30-day case fatality in critically ill COVID-19 patients with BALF-positive VAP. Table S5: multivariable analysis of independent predictors of 30-day case fatality in critically ill COVID-19 patients with BALF-positive VAP*.
