# Supplementary material for: Incidence and Prognosis of Ventilator-Associated Pneumonia in Critically Ill Patients with COVID-19: A Multicenter Study
Source: J Clin Med. 2021 Feb 3;10(4):555. doi: 10.3390/jcm10040555 (PMC7913191; doi:10.3390/jcm10040555)
Supplement: Supplementary file 1 [file jcm-10-00555-s001.pdf]

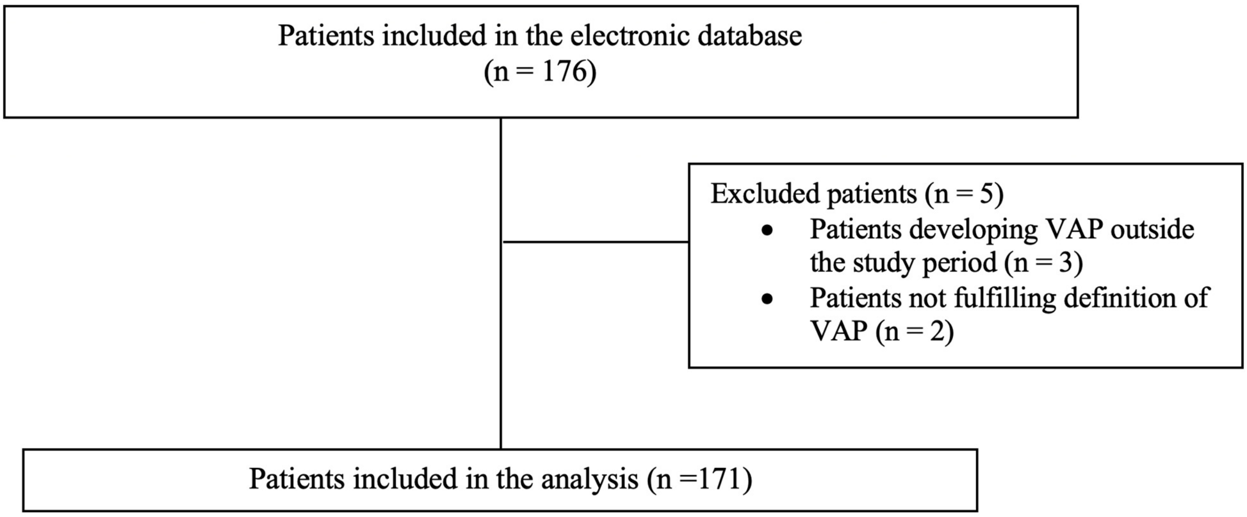

Figure 1. legend. VAP, ventilator-associated pneumonia.

Table S1. List of participating centres.

Center	No. of participating ICU (number of beds)
Ospedale Policlinico San Martino – IRCCS, Genoa*	2 (39)
Azienda Sanitaria Universitaria Giuliano-Isontina (ASUGI), Trieste	1 (16)
Cotugno Hospital, AORN dei Colli, Naples, Italy	2 (24)
Policlinico S. Orsola Hospital, Bologna, Italy	1 (60)
Maggiore Hospital, Bologna, Italy	1 (10)
Bellaria Hospital, Bologna, Italy	1 (26)
Azienda Ospedaliera Universitaria Pisana. Pisa, Italy	1 (50)
Policlinic Hospital, Modena	1 (33)
Città della Salute e della Scienza Hospital, Turin, Italy	1 (24)

* coordinating center.

Table S2. Isolates from BALF cultures.

Isolate/s	No. of patients 77 (100)
Pseudomonas aeruginosa	19 (25)
Staphylococcus aureus	13 (17)
Klebsiella pneumoniae	9 (12)
Acinetobacter spp.	6 (8)
Enterobacter aerogenes	4 (5)
Serratia marcescens	3 (4)
Klebsiella oxytoca	2 (3)
Acinetobacter spp. plus *Klebsiella pneumoniae*	2 (3)
Staphylococcus aureus plus *Stenotrophomonas maltophilia*	2 (3)
Staphylococcus aureus plus *Klebsiella pneumoniae*	2 (3)
Pseudomonas aeruginosa plus *Stenotrophomonas maltophilia*	2 (3)
Citrobacter spp.	1 (1)
Chryseobacterium indologenes	1 (1)
Escherichia coli	1 (1)
Haemophilus influenzae	1 (1)
Morganella morganii	1 (1)
Pseudomonas aeruginosa plus *Escherichia coli*	1 (1)
Pseudomonas aeruginosa plus *Klebsiella pneumoniae*	1 (1)
Pseudomonas aeruginosa plus *Proteus* spp.	1 (1)
Pseudomonas aeruginosa plus *Serratia marcescens*	1 (1)
Staphylococcus aureus plus *Enterobacter aerogenes*	1 (1)
Pseudomonas aeruginosa plus *Acinetobacter* spp. plus *Enterobacter aerogenes*	1 (1)
Pseudomonas aeruginosa plus *Klebsiella pneumoniae* plus *Proteus* spp.	1 (1)
Stenotrophomonas maltophilia plus *Enterobacter aerogenes* plus *Proteus* spp.	1 (1)

Table S3. Descriptive comparison of the demographic and clinical characteristics of patients with and without BALF culture.

Variable	BALF culture available* 79 (46)	No BALF culture available 92 (54)	*p***
**Demographics**			
Age in years, median (IQR)	62 (57-69)	66 (57-72)	0.259
Male gender	62 (78)	75 (82)	0.619
BMI in kg/m^2, median (IQR)	27 (24-30)	28 (26-30)	0.357
**Baseline comorbidities**			
Diabetes mellitus	18 (23)	21 (23)	0.095
Hypertension	55 (70)	54 (59)	0.138
Smoking/respiratory disease	11 (14)	15 (16)	0.666
End-stage renal disease	4 (5)	6 (7)	0.754
Moderate/severe liver failure	3 (4)	0 (0)	0.097
Neurologic disease	6 (8)	0 (0)	0.009
Solid cancer	2 (3)	9 (10)	0.054
Hematological malignancy*	1 (1)	3 (3)	0.625
HIV infection	0 (0)	0 (0)	-
**Previous antibiotic therapy**			
Any*	75 (95)	87 (95)	1.000
Semisynthetic penicillins	33 (42)	43 (47)	0.515
Cephalosporins	44 (56)	44 (48)	0.305
Carbapenems	10 (13)	15 (16)	0.501
Polymyxins	0 (0)	2 (2)	0.500
Glycopeptides	3 (4)	7 (8)	0.344
Oxazolidinones	13 (17)	25 (27)	0.093
Macrolides	27 (34)	51 (55)	0.005
Fluoroquinolones	3 (4)	2 (2)	0.663
Aminoglycosides	0 (0)	0 (0)	-
**Previous anti-inflammatory therapy**			
Steroids	57 (72)	51 (55)	0.024
NSAIDs	34 (43)	6 (7)	<0.001
Chloroquine/Hydroxychloroquine	75 (95)	84 (91)	0.354
Anti-IL-6 receptor monoclonal antibodies	45 (57)	64 (70)	0.087
Monoclonal IL-1 receptor antagonists	3 (4)	2 (2)	0.663
**VAP characteristics**			
Days of invasive ventilation before VAP, median (IQR)	10 (4-17)	9 (5-13)	0.821
SOFA score at VAP onset, median (IQR)	7 (5-9)	6 (3-9)	0.079

Tracheostomy before VAP	30 (38)	19 (21)	0.013
Presence of septic shock at VAP onset	47 (60)	33 (36)	0.002
Presence of ARDS at VAP onset	53 (67)	79 (86)	0.004
Presence of AKI at VAP onset	25 (32)	16 (17)	0.030
Need for hemodialytic therapy at VAP onset	9 (11)	8 (9)	0.557
Need for ECMO at VAP onset*	5 (6)	8 (9)	0.560
Presence of coagulative disorders at VAP onset			0.094
None	57 (72)	75 (82)	
Thrombotic	10 (13)	10 (11)	
Hemorrhagic	12 (15)	5 (5)	
Both	0 (0)	2 (2)	
BSI at VAP onset	45 (57)	31 (34)	0.002
**VAP treatment**			
Antibiotic treatment within 24 h of VAP onset	66 (84)	59 (64)	0.004
IgM-enriched intravenous immunoglobulins	5 (6)	5 (5)	1.000
Cytokine blood filtration	16 (20)	2 (2)	<0.001
**Outcome**			
30-day case fatality	33 (42)	45 (49)	0.350

Results are reported as number of patients (%) unless otherwise indicated. AKI, acute kidney injury; ARDS; acute respiratory distress syndrome; BALF, bronchoalveolar lavage fluid; BMI, body mass index; BSI, bloodstream infection; COVID-19, coronavirus disease 2019; ECMO, extracorporeal membrane oxygenation; HIV, human immunodeficiency virus; ICU, intensive care unit; IQR, interquartile range; NSAIDs, nonsteroidal anti-inflammatory drugs; SOFA, sequential organ failure assessment; VAP, ventilator-associated pneumonia. * Only 2/79 (2.5%) patients had negative BALF culture, whereas 77/79 (97.5%) had positive BALF culture results. ** Descriptive comparison by means of chi-square or Fisher exact tests for categorical variables, as appropriate, and Mann-Whitney U test for continuous variables.

Table S4. Univariable analysis of factors associated with 30-day case fatality in critically ill COVID-19 patients with BALF-positive VAP.

Variable	Non-survivors 32 (42)	Survivors 45 (58)	OR (95% CI)	*p*
**Demographics**				
Age in years, median (IQR)	63 (58-72)	63 (57-69)	1.01 (0.97-1.06)	0.592
Male gender	23 (72)	36 (80)	0.64 (0.22-1.85)	0.408
BMI in kg/m^2, median (IQR)	26 (24-33)	28 (25-30)	1.00 (0.99-1.02)	0.756
**Baseline comorbidities**				
Diabetes mellitus	9 (28)	8 (18)	1.81 (0.61-5.36)	0.284
Hypertension	24 (75)	30 (67)	1.50 (0.55-4.13)	0.432
Smoking/respiratory disease	9 (28)	12 (27)	1.08 (0.39-2.97)	0.887
End-stage renal disease	2 (6)	1 (2)	2.93 (0.25-33.82)	0.388
Moderate/severe liver failure	2 (6)	1 (2)	2.93 (0.25-33.82)	0.388
Neurologic disease	1 (3)	4 (9)	0.33 (0.04-3.11)	0.333
Solid cancer	1 (3)	1 (2)	1.42 (0.09-23.57)	0.807
Hematological malignancy*	1 (3)	0 (0)	4.33 (0.22-640.80)	0.333
HIV infection	0 (0)	0 (0)	-	-
**Previous antibiotic therapy**				
Any*	28 (88)	45 (100)	0.07 (0.00-0.69)	0.019
Semisynthetic penicillins	15 (47)	19 (42)	1.21 (0.49-3.01)	0.685
Cephalosporins	13 (41)	29 (64)	0.38 (0.15-0.96)	0.041
Carbapenems	3 (9)	4 (9)	1.06 (0.22-5.10)	0.942
Polymyxins	0 (0)	0 (0)	-	-
Glycopeptides	1 (3)	1 (2)	1.42 (0.09-23.57)	0.807
Oxazolidinones	7 (22)	5 (11)	2.24 (0.64-7.83)	0.207
Macrolides	11 (34)	17 (38)	0.86 (0.34-2.22)	0.760
Fluoroquinolones	2 (6)	1 (2)	2.93 (0.25-33.82)	0.388
Aminoglycosides	0 (0)	0 (0)	-	-
**Previous anti-inflammatory therapy**				
Steroids	21 (66)	34 (76)	0.62 (0.23-1.67)	0.344
NSAIDs	9 (28)	24 (53)	0.34 (0.13-0.90)	0.030
Chloroquine/Hydroxychloro-quine	30 (94)	43 (96)	0.70 (0.09-5.23)	0.726

Anti-IL-6 receptor monoclonal antibodies	15 (47)	30 (67)	0.44 (0.17-1.12)	0.085
Monoclonal IL-1 receptor antagonists	2 (6)	1 (2)	2.93 (0.25-33.82)	0.388
**VAP characteristics**				
Days of invasive ventilation before VAP, median (IQR)	9 (4-18)	12 (5-17)	0.99 (0.93-1.04)	0.617
SOFA score at VAP onset, median (IQR)	7 (5-11)	7 (5-8)	1.14 (0.98-1.33)	0.082
Tracheostomy before VAP	7 (22)	21 (47)	0.32 (0.12-0.89)	0.029
Presence of septic shock at VAP onset	25 (78)	21 (47)	4.08 (1.47-11.35)	0.007
Presence of ARDS at VAP onset	31 (97)	21 (47)	35.43 (4.45-282.36)	0.001
Presence of AKI at VAP onset	13 (41)	11 (24)	2.12 (0.79-5.63)	0.134
Need for hemodialytic therapy at VAP onset	5 (16)	3 (7)	2.59 (0.57-11.75)	0.217
Need for ECMO at VAP onset*	4 (13)	0 (0)	14.37 (1.45-1931.17)	0.019
Presence of coagulative disorders at VAP onset				0.426
None	22 (69)	34 (76)	Ref.	
Thrombotic	3 (9)	6 (13)	0.73 (0.18-3.42)	
Hemorrhagic	7 (22)	5 (11)	2.16 (0.61-7.68)	
Both	0 (0)	0 (0)	-	
Microorganisms isolated from BALF				0.514
Pseudomonas aeruginosa	9 (28)	18 (40)	0.64 (0.22-1.86)	
Staphylococcus aureus	9 (28)	9 (20)	1.29 (0.40-4.09)	
Other than *P. aeruginosa* and *S. aureus*	14 (44)	18 (40)	Ref.	
Isolation of resistant organisms from BALF				0.876
MRSA	4 (13)	4 (9)	1.44 (0.32-6.54)	
Carbapenem-resistant Gram-negative bacteria	10 (31)	15 (33)	0.96 (0.35-2.62)	
None of them	18 (56)	26 (58)	Ref.	
BSI at VAP onset	17 (53)	26 (58)	0.83 (0.33-2.06)	0.685

**VAP treatment**				
Antibiotic treatment within 24 h of VAP onset	26 (81)	38 (84)	0.80 (0.24-2.65)	0.713
Appropriate antibiotic treatment within 24 h of VAP onset	19 (59)	26 (58)	1.07 (0.43-2.68)	0.889
IgM-enriched intravenous immunoglobulins	3 (9)	2 (4)	2.22 (0.35-14.15)	0.397
Cytokine blood filtration	6 (19)	9 (20)	0.92 (0.29-2.91)	0.891

Results are reported as number of patients (%) unless otherwise indicated. AKI, acute kidney injury; ARDS; acute respiratory distress syndrome; BALF, bronchoalveolar lavage fluid; BMI, body mass index; BSI, bloodstream infection; CI, confidence intervals; COVID-19, coronavirus disease 2019; ECMO, extracorporeal membrane oxygenation; HIV, human immunodeficiency virus; ICU, intensive care unit; IQR, interquartile range; NSAIDs, nonsteroidal anti-inflammatory drugs; OR, odds ratio; SOFA, sequential organ failure assessment; VAP, ventilator-associated pneumonia. * Standard logistic regression model not converging. Results for these variables are from univariable logistic regression models with Firth's correction.

Table S5. Multivariable analysis of independent predictors of 30-day case fatality in critically ill COVID-19 patients with BALF-positive VAP*.

Variable	OR (95% CI)	*p*
Any previous antibiotic therapy	0.51 (0.00-8.20)	0.673
Previous cephalosporins	0.38 (0.09-1.44)	0.155
Previous NSAIDs	1.59 (0.39-7.19)	0.519
Previous anti-IL-6 receptor monoclonal antibodies	0.52 (0.12-1.95)	0.332
Tracheostomy before VAP	0.30 (0.06-1.15)	0.080
SOFA score at VAP onset	0.94 (0.74-1.19)	0.597
Presence of septic shock at VAP onset	3.69 (0.95-17.49)	0.060
Presence of ARDS at VAP onset	44.09 (5.97-694.29)	<0.001**
Need for ECMO at VAP onset	18.1 (0.81-3366.72)	0.070
Antibiotic treatment within 24 h of VAP onset	0.79 (0.08-7.56)	0.832
Appropriate antibiotic treatment within 24 h of VAP onset	0.38 (0.07-1.74)	0.214

ARDS; acute respiratory distress syndrome; CI, confidence intervals; COVID-19, coronavirus disease 2019; ECMO, extracorporeal membrane oxygenation; NSAIDs, nonsteroidal anti-inflammatory drugs; OR, odds ratio; SOFA, sequential organ failure assessment; VAP, ventilator-associated pneumonia. * Standard logistic regression model not converging. Results are from multivariable logistic regression with Firth's correction. ** $p < 0.05$.
